# Illumina Miseq platform analysis caecum bacterial communities of rex rabbits fed with different antibiotics

**DOI:** 10.1186/s13568-016-0273-1

**Published:** 2016-10-21

**Authors:** Fuqin Zou, Dong Zeng, Bin Wen, Hao Sun, Yi Zhou, Mingyue Yang, Zhirong Peng, Shuai Xu, Hesong Wang, Xiangchao Fu, Dan Du, Yan Zeng, Hui Zhu, Kangcheng Pan, Bo Jing, Ping Wang, Xueqin Ni

**Affiliations:** 1 Animal Microecology Institute, College of Veterinary Medicine, Sichuan Agricultural University, Chengdu, 611130 Sichuan China; 2 Key Laboratory of Animal Disease and Human Health of Sichuan Province, Chengdu, 611130 Sichuan China; 3 Sichuan Academy of Grassland Science, Chengdu, 611731 Sichuan China

**Keywords:** Illumina Miseq platform, Rex rabbit, Chlortetracycline, Colistin sulfate, Zinc bacitracin, Caecum microflora

## Abstract

Antibiotics have been widely used for the prevention and the treatment of diseases to humans and animals, and they have fed additives for agricultural animals to promote growth. However, there is a growing concern over the practice due to its side effects on intestinal microbial communities which plays a vital role in animals’ health. To investigate the effect of antibiotics on the bacterial population of the caecum in rex rabbits, 80 rex rabbits were randomly divided into four groups: control group (B, basal diet), chlortetracycline group (C, 50 mg/kg), colistin sulfate group (S, 20 mg/kg) and zinc bacitracin group (Z, 40 mg/kg). Caecum microbial communities of rex rabbits from the four groups were analyzed through Illumina Miseq platform after being fed 28 days. The results showed that most obtained sequences belongs to Firmicutes followed by Bacteroidetes, and the ratio of Bacteroidetes*/*Firmicutes in C group (42.31 %) was higher than that in Z group (21.84 %). Zinc bacitracin supplementation caused a significant decreased of the Proteobacteria phylum and *Lactobacillus* spp. (*P* < 0.05), while the *Lactobacillus* spp. significantly increased in S group (*P* < 0.05). In addition, *Ruminococcus* spp., especially *Ruminococcus albus* were the predominant bacterial species found in both S and Z groups. The proportion of *Coprococcus* spp. significantly increased in Z group (*P* < 0.05). These findings suggested that the antibiotics used may cause significant changes in the caecum microbiota of rex rabbits, and we also found C group had a similarity caecum bacteria structure with B group which was probably due to the high levels of chlortetracycline resistance.

**Electronic supplementary material:**

The online version of this article (doi:10.1186/s13568-016-0273-1) contains supplementary material, which is available to authorized users.

## Introduction

The rex rabbit is an important small herbivorous mammal which is widely raised for the fur and meat production. As a hindgut fermentation animal, the rabbit caecum has a relatively large size and rich bacterial communities, which are closely linked with gut health and digestive efficiency (Zhu et al. 2015; Bäuerl et al. 2014). So, these physiological characteristics of the rabbit intestinal microorganisms are getting more and more attention. Caecal microorganisms play an important role in intestinal health and host function, which are mainly used to improve the development of the immune system, such as improving the immune function of the intestinal tract (Chung et al. 2012), increasing fat storage (Cho et al. 2012; Liou et al. 2013), and improving the rabbit digestion and absorption capacity with the utilize of protein, energy and other synthesize nutrient (Lepage et al. 2013; Zhang et al. 2013). Therefore, the microbial community structure of the rabbit intestinal tract is particularly important, which is closely related to the health of the host. Intestinal health of domestic rabbits is quietly delicate and any disruption of the digestive process may result in gastrointestinal diseases (Zhu et al. 2015), and the development of diseases is generally associated with the changes in intestinal microflora. In the rabbit breeding industry, antibiotic additives are also added in animal diets to achieve the purpose of promoting the growth and preventing the gastrointestinal disease.

Antimicrobial agents have been widely used in feeding since the 1950s to improve the feed efficiency and animal growth through the modulation of the gut microbiota and host immune response (Feighner and Dashkevicz 1987) as well as to reduce morbidity and mortality due to clinical and/or subclinical disease (Niewold 2007). However, due to the abuse of antibiotics, a series of problems break out. For example, the increasing resistance of bacteria poses a health risk to human. The use of antibiotics as growth promoters (AGPs) in animal feeds has been banned in European Union (EU) since 2006. However, considering the ban of antibiotics may lead to an increase of safety risks in animal source food (Poduval et al. 2000; Jones 2000), the use of some AGPs in animal feeds is still allowed in many countries. Antibiotics not only reduce the risk of potential infectious diseases, but also may affect the symbiotic bacterial population of the digestive tract and improve the utilization of nutrition in growing rabbits (Abecia et al. 2007). Dietary supplementation of antibiotics has been demonstrated to influence gut microbiota by enhancing metabolic capacities, improving digestion and absorption of nutrients (Pedersen et al. 2013). Currently, people have focused on the impact of antibiotics on intestinal microflora and the role of the intestinal microbiota in animal health, production, and product safety (Torok et al. 2011). It is necessary to guide the use of antibiotics feeds from the perspectives of the intestinal bacterial community.

In this study, we have chosen three kinds of commonly used antibiotics in rabbit production, namely, colistin sulfate, zinc bacitracin and chlortetracycline. We used Illumina Miseq platform to characterize the rex rabbits caecum microbial communities structure shifts induced by the three antibiotics. The experiment is designed to evaluate the effect of antibiotics on rex rabbit intestinal microflora and we hope to extend our knowledge about these, as well as to provide guidance of the reasonable use and to avoid the abuse of the three antibiotics in rex rabbits.

## Materials and methods

### Experimental design

Rex rabbits of mixed sex weaning at 28th day followed 7 days of acclimation in separated cages under the same temperature (25 ± 2 °C controlled by automatic heating and ventilation devices) with access to customized fodder without antibiotics and water ad libitum. A total of 80 rex rabbits were randomly allotted into one control and three treatments, each group with 5 replicates of 4 rex rabbits. The dietary treatments were as follows: basal control diet (B group); basal diet + 50 mg/kg chlortetracycline (C group); basal diet + 20 mg/kg colistin sulfate (S group); basal diet + 40 mg/kg zinc bacitracin (Z group). The basal diets were formulated according to the NRC (Table 1). Over the entire experimental period (day 0–28), all animals were housed in a temperature- and humidity- controlled room with a 12 h light/dark cycle that ensured all rex rabbits lived in a consistent growth environment and all were allowed free access to food and water.

**Table 1:** Composition and nutrient levels of the complete diets

Ingredients	Content/%	Nutrient levels	Content/%
Two grad corn	15.00	ME (MJ/Kg)	10.2
Middlings	8.00	Crude Protein	16.57
Wheat bran	16.00	Crude Fiber	14.82
Chaff 37	10.00	Ca	0.95
Alfalfa meal	32.50	Total P	0.68
Soybean meal	14.00	Lysine	0.86
Soycomil	1.00	Methionine	0.38
Calcium hydrogen phosphate	1.00	Cystine	0.24
Limestone	0.50		
Bentonite	0.80		
NaCl	0.40		
mineral additive	0.50		
Lysine	0.10		
Methionine	0.15		
Chinese multivitamin	0.05		
Total	100		

Feed nutrient levels according to the Chinese Feed Ingredients and Nutritional Value Table of the Twenty-fourth Edition in 2013 with EXCEL2007 were calculated to the adding proportion. The mineral additive and Chinese multivitamin followed the diet per kilogram to add: Fe 40, Cu 30, Zn 20, Mn 10, Mg 20 mg, VA 900, VD 600, VE 60 IU.

### Sample collection

At the end of the experiment, one healthy rex rabbit from each replicate was randomly selected and euthanized by cervical dislocation, while the dissected and the caecum were kept on ice immediately. The caecum surface was sterilized with 70 % ethanol. A longitudinal incision was made of a scalpel and the edges were far apart, and the luminal contents were collected into a sterile tube. Samples were immediately transferred into liquid nitrogen for temporary storage and stored at −80 °C until further study.

### DNA extraction

Total DNA was extracted from the caecum content of randomly selected rex rabbits (n = 5, each group) by using E.Z.N.A.*™* stool DNA kit (Omega Bio-Tek, Doraville, USA) according to the manufacturer’s instructions. For a better analysis of Gram-positive bacteria DNA, the second incubation at 95 °C for 10 min was performed following the initial incubation at 70 °C for 13 min in the protocol. The isolated DNA was eluted in 100 μL of elution buffer, and the concentration was determined by a Nano Drop spectrophotometer (Nano Drop Technologies, Wilmington, DE, USA). Finally, DNA was stored at −80 °C before the further analysis.

### PCR amplification and high-throughput sequencing

The V4 region of the 16S rDNA gene was amplified with the primers 515F/806R (515F:5′-XXXXXXGTGCCAGCMGCCGCGGTAA-3′;806R:5′-XXXXXXGGACTACHVGGGTWTCTAAT-3′) by using MyCycler*™* Thermal Cycler (Bio-Rad Laboratories, USA), and the 5′ terminus of each primer contained a 6-bp error-correcting barcode sequence to tag specific samples (Zeng et al. 2015). The PCR was conducted in triplicate for each sample of the reaction mixture (30 μL) containing 15 μL of Phusion^®^ High-Fidelity PCR Master Mix (New England Biolabs (Beijing) LTD., China), 1.5 μL of each primers (BGI Tech Solutions Co., Ltd. (BGI-Tech), China) (2 μM), 10 μL of template DNA (1 ng/μL) and 2 μL of sterile deionized water. PCR conditions were as follows: initial denaturing step at 98 °C for 1 min (1 cycle), followed by 35 cycles of 98 °C for 10 s, 50 °C for 60 s, 72 °C for 30 s, and a final extension of 10 min at 72 °C. Subsequently, PCR products of each sample were detected by using a 1.0 % agarose gel (Thompson et al. 2002) and purified by using a GeneJET Gel Extraction Kit (Thermo Scientific, USA). Sequencing was performed at the Novogene Bioinformatics Technology Co., Ltd with an Illumina MiSeq platform according to protocols described by previous studies (Caporaso et al. 2012).

Following the manufacturer’s recommendations, a sequencing library was generated by using NEB Next^®^ Ultra*™* DNA Library Prep Kit for Illumina (New England Biolabs (Beijing) LTD., China) and index codes were added. The library quality was assessed on the Qubit@ 2.0 Fluorometer (Thermo Scientific, USA) and Agilent Bioanalyzer 2100 system. The last step, the library was sequenced on an Illumina Miseq platform.

### Bioinformatics analyses

The sequences were analyzed with the QIIME (Caporaso et al. 2010) software package along with custom Perl scripts to analyze alpha- (within samples) and beta- (among samples) diversity. Raw reads were filtered by QIIME quality filters (removal of chimeric, contamination and low quality sequences in the raw reads) in order to obtain the pure sequencing data. Operational taxonomic units (OTUs) were picked by using de novo OTUs picking protocol with a 97 % identity threshold, then a representative sequence was picked for each OTU and using the RDP (http://rdp.cme.msu.edu) classifier (Wang et al. 2007) to annotate taxonomic information for each representative sequence. Alpha diversity calculation includes four metrics (http://www.mothur.org/wiki/): Chao1, Observed species, Shannon and Simpson indices. Rarefaction curves were generated based on these four metrics. QIIME calculated both weighted and unweighted unifrac, which was phylogenetic measures of beta diversity. Beta diversity included both unweighted and weighted unifrac distances, and these distances were visualized by principal coordinate analysis (PCoA) (Lozupone and Knight 2005). We compared overall samples between inter-group and intra-groups composition that used pairwise multiresponse permutation procedure (MRPP). MRPP is a nonparametric procedure to test the hypothesis of no difference between two or more groups of entities and can analyze comparisons between all groups. The MRPP calculate the average intra-group distance between samples and compares it with the average inter-group distances, providing a measure of dissimilarity by means of a “delta score” (Abella et al. 2003). The raw read sequences have been deposited at the National Center for Biotechnology Information (NCBI) Sequence Read Archive (http://www.ncbi.nlm.nih.gov/sra) with study accession number SRP068345.

### Statistical analysis

One-way analysis of variance (ANOVA) was used to analyze differences in caecum microflora of rex rabbits between the control group and antibiotics groups by SPSS 19.0 software (SPSS Inc., Chicago, Illinois, USA). Significant was reported at *P* < 0.05.

## Results

### Illumina MiSeq derived metadata

**Fig. 1:**
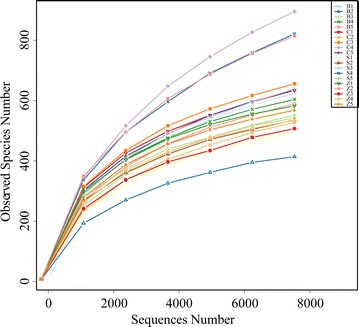
Rarefaction curves represent the OTUs of per sample bacterial diversity in the caecum of rex rabbits

Overall, 20 samples of contents were used to assess the effects of dietary antibiotic supplementation (chlortetracycline or colistin sulfate or zinc bacitracin) on caecum microflora of rex rabbits. We performed a high-throughput sequencing on the Illumina MiSeq platform and results revealed a total of 962,256 reads, which had passed all quality filters under 97 % identity conditions to obtained a total of 2562 species classification OTUs. On average, there were 128 OTUs for each sample (Additional file 1: Table S1). The rarefaction curves (OTUs at the 97 % identity) were shown on Fig. 1.

**Fig. 2:**
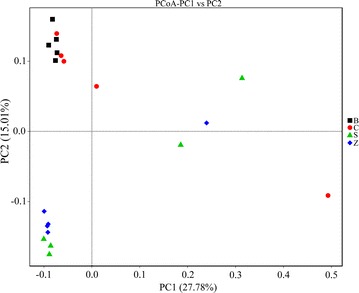
The PCoA analysis of the rex rabbits caecum contents. The *horizontal* coordinate represents one principal component and the *longitudinal* coordinates represents another principal component. The percentage represents contribution of principal component to the difference of samples. Each symbol represents each gut microbiota. *Black* represents of the B group, *red* represents of the C group, *green* represents of the S group, *blue* represents of the Z group

Observed species and Chao1 reflect the richness of species within a single sample, while Shannon and Simpson indexes represent microbial diversity. As shown in Table 2, Observed species, Chao1, Shannon and Simpson indexes in the three antibiotic groups were higher than that in control group, whereas there was no statistical difference in alpha diversity among the four groups (*P* > 0.05). Bacterial communities were clustered by using PCoA of unweighted unifrac distance matrices (Fig. 2). It was surprising that rex rabbits fed with C and B groups had higher similarities bacterial members than S and B, and Z and B through clustering closely on the two-dimensional PCoA plot. Meanwhile, these were consistent with the results of MRPP analysis (Table 3).

**Table 2:** Richness and diversity estimation for caecum bacterial populations based on alpha diversity analysis

Samples name	Observed species	Chao1	Shannon	Simpson
B1	625	848.350	6.746	0.972
B2	414	495.759	5.525	0.936
B3	551	677.378	6.356	0.954
B4	604	783.033	7.080	0.978
B5	589	762.532	6.245	0.934
C1	634	821.736	7.487	0.988
C2	528	698.200	5.950	0.939
C3	656	877.796	7.277	0.980
C4	895	1303.983	6.693	0.952
C5	635	838.029	7.162	0.980
S1	494	630.988	6.432	0.963
S2	536	681.283	7.023	0.980
S3	639	841.736	6.753	0.957
S4	821	1183.601	7.345	0.982
S5	542	634.221	7.288	0.987
Z1	582	691.698	7.399	0.987
Z2	814	1221.500	7.515	0.983
Z3	507	625.168	6.122	0.947
Z4	535	640.061	6.906	0.978
Z5	568	676.083	7.068	0.978

**Table 3:** MRPP analysis microbial community structure among groups

Group	A	Observed-delta	Expected-delta	Significance
B–C	0.01415	0.5675	0.5757	0.347
B–S	0.06663	0.5807	0.6221	0.008*
B–Z	0.03992	0.5153	0.5367	0.007*
C–S	0.06086	0.589	0.6272	0.012*
C–Z	0.03771	0.5236	0.5441	0.107
S–Z	0.04845	0.5368	0.5641	0.017*

* Mean the significantly different between groups (*P* < 0.05)

The A values greater than 0 indicated that the difference in intra-group is greater than that in inter-group, but less than 0 represented that the difference in inter-group is greater than that in intra-group. Observe-delta value is smaller which indicates that the difference is small within groups, and the Expect-delta value is greater which indicates that the difference is great between groups. The significance value below 0.05 indicate that the difference is significant.

### Differences in bacterial communities between antibiotics and control groups

**Fig. 3:**
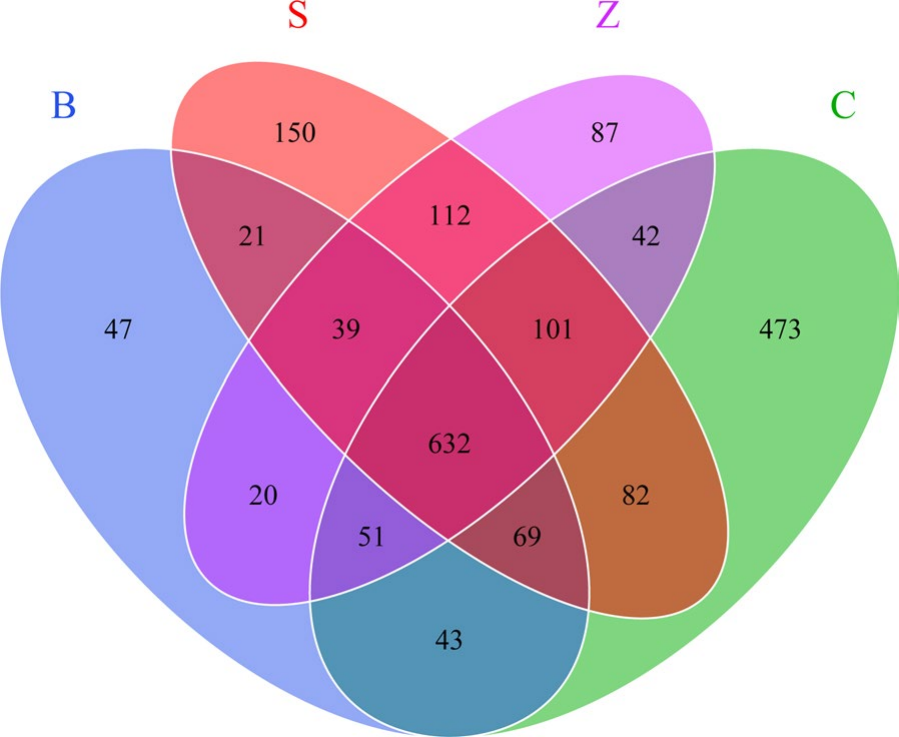
Venn diagram summarizing the numbers of common and unique OTUs (3 % distance level) among the four groups. Each *circle* represents a set of samples, the group between the circle and circle overlapping part digital represents of the common OTUs and there is no overlapping part represents unique OTUs in each group. *Blue* represents the B group; *green* represents the C group; *rose* represents the S group; *purple* represents the Z group

A venn diagram displayed that a total of 632 OTUs were identified as constituting core bacterial OTUs in the four groups (Fig. 3). The number of unique OTUs in each group was 47 (B), 473 (C), 150 (S) and 87 (Z), respectively. The OTUs shared in antibiotic groups and control group were 795 (B&C), 761(B&S), 742 (B&Z). These results together with PCoA analysis suggested that rex rabbits from B and C groups may have similar bacterial communities in their caecum.

**Fig. 4:**
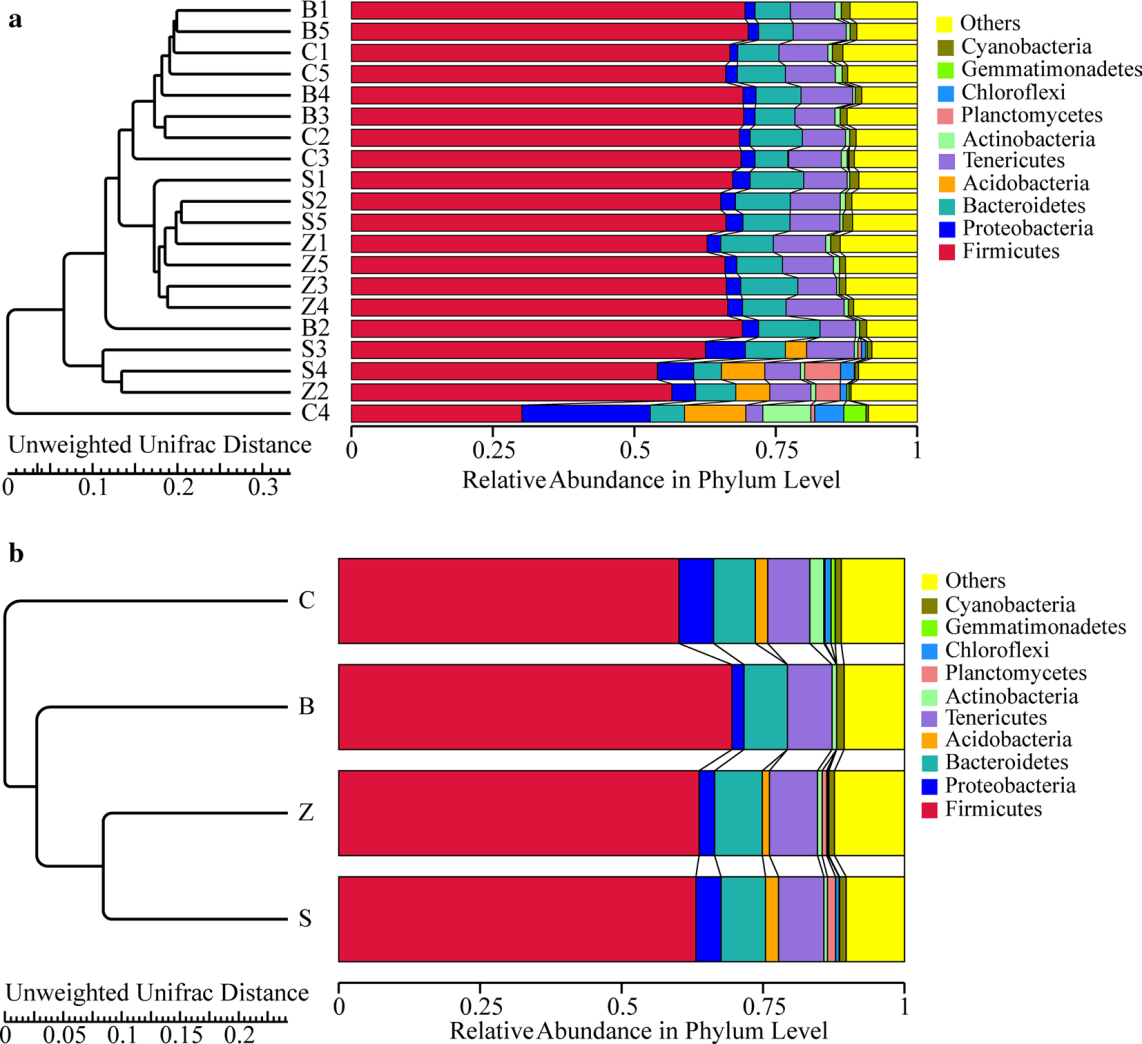
Relative abundance of the main bacterial communities found in each samples (**a**) and groups (**b**) in the rex rabbits caecum assigned at the phylum level. The *horizontal* coordinates is expresses the relative abundance and the *longitudinal* coordinate represents the samples (**a**) or groups (**b**) name. Each *bar* represents the average relative abundance of each bacterial taxon within a sample or group. The top 10 abundant taxa were shown

At the phylum level, a total of 26 bacteria forming by representatives of 5 phyla were identified (Additional file 1: Table S2), including Firmicutes, Bacteroidetes, Verrucomicrobia, Proteobacteria and Tenericutes. The majority of obtained sequences belonged to Firmicutes, which followed by Bacteroidetes (Fig. 4), and these two phyla constituted roughly 83 % of the caecum microbiota. In comparison with the B group, dietary supplementation with colistin sulfate and zinc bacitracin increased Firmicutes but decreased Bacteroidetes, while a decreased of Firmicutes and an increment of Bacteroidetes were observed in chlortetracycline supplemented of rex rabbits diets (Firmicutes B: 64.31 %, C: 58.21 %, S: 66.05 %, Z: 69.61 %. Bacteroidetes B: 20.44 %, C: 24.63 %, S: 17.47 %, Z: 15.20 %). The ratio of Bacteroidetes/Firmicutes in B, C, S and Z groups was 31.78, 42.31, 26.45 and 21.84 %, respectively. In comparison to the B group, dietary supplementation with chlortetracycline increased Bacteroidetes/Firmicutes ratio, while were a decreased in S and Z groups. When compared with the B group, a significant decreased of Proteobacteria and *Deltaproteobacteria* were observed in Z group (*P* < 0.05). Moreover, the proportion of *Deltaproteobacteria* in S group was significantly lower than that in B group (*P* < 0.05).

**Fig. 5:**
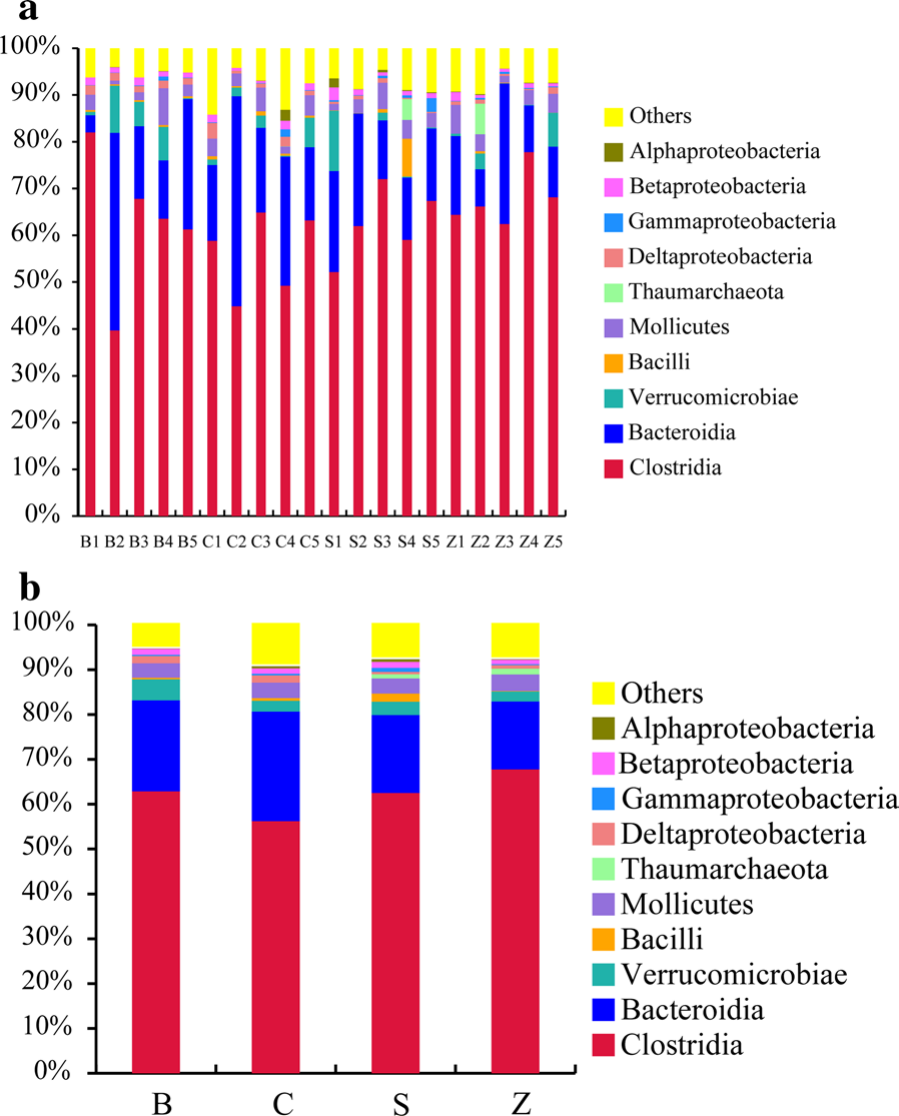
Relative abundance of the main bacterial communities found in each samples (**a**) and groups (**b**) in the rex rabbits caecum assigned at the class level. The *horizontal* coordinate represents the samples (**a**) or groups (**b**) name and the *longitudinal* coordinates is expresses the relative abundance. Each *bar* represents the average relative abundance of each bacterial taxon within a sample or group. The top 10 abundant taxa were shown

At class level, the relative abundance of dominant microbial species found in the four groups was shown in Fig. 5. *Clostridia* class was predominant in the four groups (B: 62.87 %, C: 56.20 %, S: 62.52 %, Z: 67.77 %). There was a significant difference in the proportion of *clostridia* between Z and C groups (*P* < 0.05). *Bacteroidia* class had a high proportion in C group, but the proportion was low in Z group compared with the B group. The proportion of *Bacilli* class in Z group was significantly lower than that in C and B groups (*P* < 0.05), and it significantly increased in S group compared with the B group (*P* < 0.05), probably due to *Lactobacillus* spp. While *Lactobacillus* spp. significantly decreased in Z group, it significantly increased in S group (*Lactobacillus* spp. B: 0.28 %, C: 0.37 %, S: 1.67 %, Z: 0.08 %) (*P* < 0.05).

**Fig. 6:**
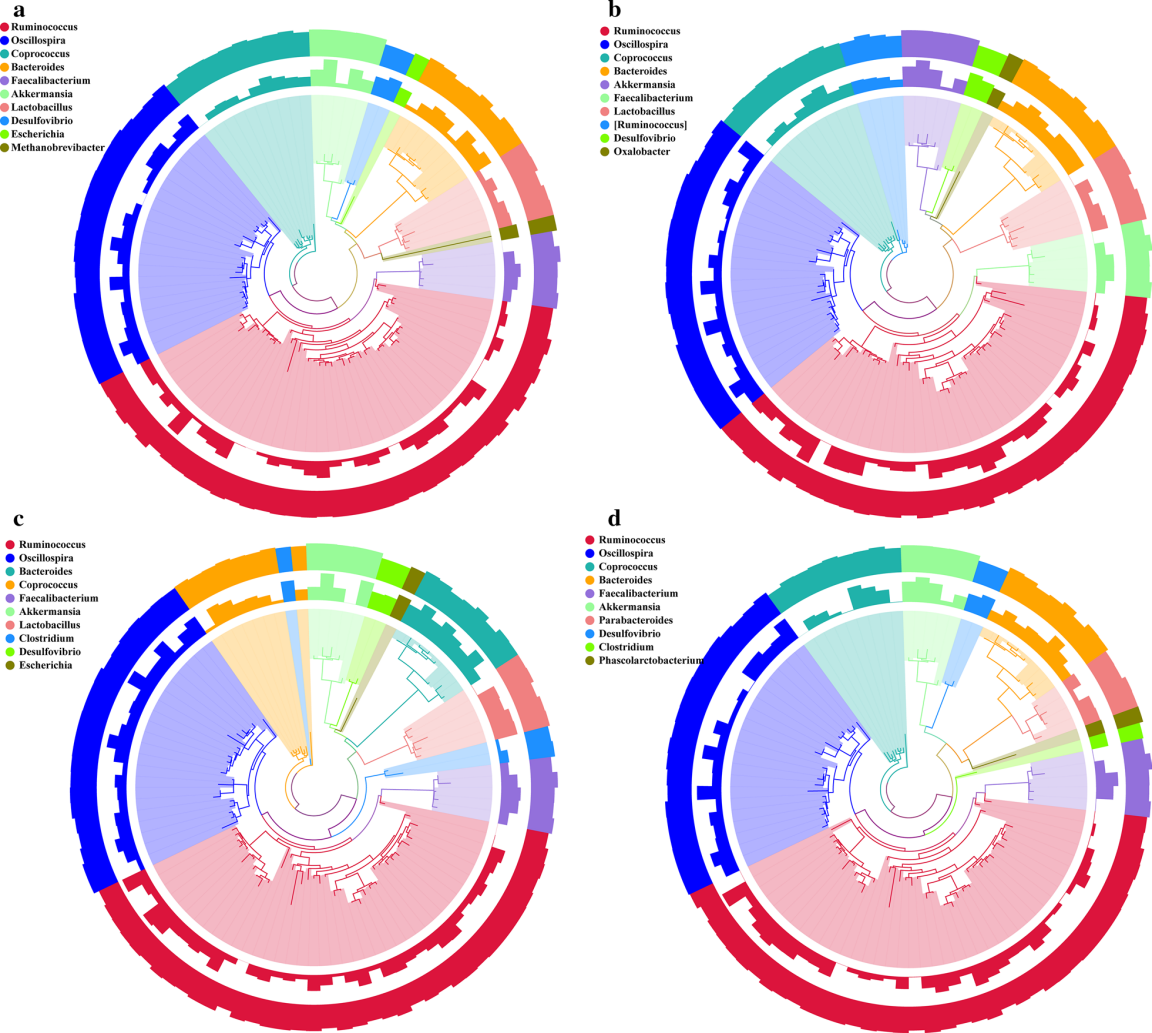
Relative abundance of the main bacterial communities found in each groups in the rex rabbits caecum assigned at the genus level. **a**–**d** Control, chlortetracycline, colistin sulfate and zinc bacitracin main bacterial communities, respectively. The *circle map* consists of three layers, from inside to outside: the first layer is the phylogenetic tree constructed by OTUs representative sequence and the *branches colors* indicate the corresponding genus name and each color represents a genus; the second layer is the relative abundance distribution of OTUs, the height of column indicates that the relative abundance of OTUs (Owing to the relative abundance across a large amount of data, in accordance with the minimum value of transformation and visualization); the three layer is the credibility of the OTUs annotation distribution, and the height of column indicates the credibility of OTUs notes

We had analyzed the lower taxonomical genus levels showing that the predominant of *Ruminococcus* spp. (Figure 6) (B: 5.02 %, C: 5.30 %, S: 5.63 %, Z: 6.04 %) and the addition of antibiotics increased *Ruminococcus* spp. richness, especially in the Z group. *Ruminococcus albus* in S and Z groups were significantly higher than that in B group (*P* < 0.05) (B: 0.13 %, C: 0.32 %, S: 1.32 %, Z: 0.39 %). *Oscillospira* spp. played a promoting role in intestinal fermentation and it increased in S and Z groups (B: 3.75 %, C: 3.84 %, S: 4.63 %, Z: 4.44 %). *Akkermansia* spp. had a high proportion of the rex rabbit caecum and previous studies were proposed to be a contributor to the maintenance of intestinal health that *Akkermansia muciniphila* in S group had a higher proportion (B: 0.071 %, C: 0.068 %, S: 1.16 %, Z: 0.066 %). This study showed that S and Z groups significantly decreased *Desulfovibrio* spp. (*P* < 0.05). *Coprococcus* proportions (B: 0.43 %, C: 0.77 %, S: 0.74 %, Z: 1.21 %) significantly increased (*P* < 0.05) and *Faecalibacterium* had an upward trend due to the addition of zinc bacitracin in diets (B: 0.39 %, C: 0.44 %, S: 0.65 %, Z: 0.92 %). The proportions of *Streptococcus* spp. and *Escherichia* spp. slightly decreased by adding zinc bacitracin, but they slightly increased in the S group. Adding chlortetracycline in diets led to the decreasing proportion of *Escherichia* spp. but *Streptococcus* spp. almost no change.

## Discussion

The rex rabbits gastrointestinal (GI) tract is colonized by a dense microflora community that is intimately connected to the overall heath and the development of its animal host. In livestock, there is a considerable interest in understanding how this community contributes to the efficient feed conversion into animal growth (Neumann and Suen 2015). Improvements in feed conversion associated with dietary supplementation with antibiotics are thought to involve GI tract microbial communities, but this connection remains poorly understood (Dibner and Richards 2005). Here, we sought to elucidate the effects of chlortetracycline, colistin sulfate and zinc bacitracin on the rex rabbits caecum bacterial communities using high-throughput sequencing on the Illumina MiSeq platform.

Subtherapeutic of antibiotics used as additives in livestock diets may shape a more complex intestinal microorganism and even accelerate the maturation of gut microflora structure. Our study found alpha diversity indices that the reflected antibiotics can improve the richness and diversity of rex rabbits caecum microorganism. While this was contradicted with the general view about antibiotics causing a reduction in bacterial diversity (Dubourg et al. 2014), Peng and Zeng et al. (2016) had found that antibiotics could increase the giant pandas faeces bacterial communities using PCR-denaturing gradient gel electrophoresis (DGGE) technology analysis, which were consistent with our results. Furthermore, our experiments in represented C group had got a higher Observed species and Chao1, which were suggested that chlortetracycline than colistin sulfate and zinc bacitracin was more likely to increased rex rabbits caecum flora abundance. The results of beta diversity analysis indicated that B and C groups had a similarity caecum bacteria structure. Furthermore, they were different from B and S, and B and Z groups samples, suggested that the effect of colistin sulfate and zinc bacitracin on rex rabbits caecum microflora were greater than chlortetracycline, which was probably due to the high levels of chlortetracycline resistance.

The phylum Proteobacteria is unstable over time among the four main phyla (Firmicutes, Bacteroidetes, Proteobacteria, and Actinobacteria) in the gut microbiota and is likely to influenced by environment, such as diets (Faith et al. 2013). Proteobacteria include many pathogenic bacteria, for instance, diarrhea caused by *Escherichia coli*, *Salmonella* and *Vibrio cholera* etc. Previous studies endorse the concept that a bloom of Proteobacteria in the gut reflects dysbiosis or an unstable gut microbial community structure or a potential diagnostic criterion for disease (Shin et al. 2015). The decreased of Proteobacteria number may induced the amount of pathogenic bacteria in the body and the host morbidity to reduce (Faith et al. 2013), which was good for the animals’ health. Moreover, our studies showed that zinc bacitracin significantly reduced rex rabbits caecum Proteobacteria. It can be seen that zinc bacitracin may play a good role in the disease prevention and be superior to chlortetracycline and colistin sulfate.

Previous studies reported that Firmicutes and Bacteroidetes are dominant phyla in mammals (e.g. rabbit) and human (Zeng et al. 2015; Panda et al. 2014; Nam et al. 2011; Gu et al. 2013; Qin et al. 2010). We also had a similar result which was found in the rex rabbits. Changes within the Bacteroidetes/Firmicutes ratio have become the focus of microbiome-associated obesity research (Ismail et al. 2010), and the increased of the ratio may contribute to the decreasing body weight (Jami et al. 2014). Furthermore, the gut microbiota of obese individuals are rich in Firmicutes and scarce in Bacteroidetes compared with lean individuals (Ley et al. 2006). It was reported that the increase on the number of the *Bacteroidia* was negatively correlated with energy intake and obesity (Furet et al. 2010). In this experiment, we compared the control group discovered that the *Bacteroidia* amounts and Bacteroidetes/Firmicutes ratio slightly increased in the chlortetracycline group rex rabbits, and chlortetracycline was probably associated with rex rabbits body weight loss.

*Lactobacillus* spp. are Gram-positive facultative anaerobic bacteria that grow better in richly nutritious environment. They are important probiotics that play an important role in the intestinal inflammation and the regulation of immune function, and they are widely used in animal production to prevent disease and improve digestion rate. Rodriguez and Nadra reported (Rodriguez and Nadra 1995), *Lactobacillus* spp. can cause low pH environments through the secretion of organic acid in the animal body, and can also secrete hydrogen peroxide, bacteriocins, butanedione to prevent and inhibit harmful bacterial invasion and colonization. Meanwhile they have a certain role in maintaining intestinal flora balance. Colistin sulfate has a strong inhibitory effect on Gram-negative bacteria (Velkov et al. 2010) and zinc bacitracin has a strong inhibitory effect on Gram-positive bacteria (Injac et al. 2008), which may be one of the reasons that colistin sulfate significantly increased and zinc bacitracin significantly decreased *Lactobacillus* spp. *Lactobacillus* spp. significantly increased in C group samples suggested that the antibiotic may promote rex rabbits towards a healthier growth, and it also showed that colistin sulfate may inhibit pathogenic bacteria invasion and colonization plays a certain role. A previous study showed that antibiotic treatment dramatically reduced the abundance of *Lactobacillus* spp. in the obese (ob/ob) mice (Cani et al. 2008), so zinc bacitracin was probably positively correlated with the growth of rex rabbit’s body weight. However, relevant information in this area is very limited and further study is still needed to determine the antibiotics through regulating intestinal microflora to improve rabbit weight.

*Ruminococcus* spp. and *Oscillospira* spp. have been detected in the rumen of several herbivores such as cattle, sheep and reindeer (Mackie et al. 2003). The members of the two genera are involved in the degradation of cellulose, the intestinal fermentation and the promotion of host growth (Gulino et al. 2013; Tims et al. 2013), and *Oscillospira* spp. have been demonstrated to be associated with fermentation caecum of rex rabbits (Zeng et al. 2015). In our experiment, the addition of antibiotics increased the proportions of *Ruminococcus* spp. and *Oscillospira* spp. Especially, colistin sulfate and zinc bacitracin significantly increased *R. albus*. According to the results we assumed that colistin sulfate and zinc bacitracin were more likely to accelerate the caecum fermentation, improve feed conversion and promote energy absorption of rex rabbits than chlortetracycline. We speculated that this change may improve the growth of rex rabbits.

*Faecalibacterium prausnitzii* and *Akkermansia* are associated with inflammatory immune regulation and the gut barrier (Kim et al. 2013; Furet et al. 2010; Louis and Flint 2007; Everard et al. 2013; Hippe et al. 2014). *Faecalibacterium prausnitzii* is one of the most abundant symbiotic anaerobic bacteria in GI tract and it is named after one of the main butyrate producers (Louis and Flint 2007). Butyrate may affect inflammatory process through the regulation of inflammatory genes (Andoh et al. 1999; Place et al. 2005). *Akkermansia* muciniphila is a Gram-negative mucin-degrading bacterium (Derrien et al. 2008). *Akkermansia* are known to feed on mucus and may indicate inflammatory processes (Everard et al. 2013). Furthermore, it has a positive correlation between *A. muciniphila* and health, and induced *A. muciniphila* expansion leds to metabolism improvement (Dao et al. 2016). Our study showed that *F. Prausnitzii* (B: 0.072 %, C: 0.058 %, S: 0.174 %, Z: 0.171 %) and *A. Muciniphila* (B: 0.071 %, C: 0.068 %, S: 1.16 %, Z: 0.066 %) increased through the addition of colistin sulfate in diets. *F. Prausnitzii* also increased in the zinc bacitracin group. So, it implied that colistin sulfate and zinc bacitracin probably can modulate intestinal inflammation on rex rabbits, which is still needed in our further research.

Previous work reported that the major butyrate producers *coprococcus* spp. (Jost et al. 2014) show low in mice exposed to social disruption stress and correlated to stressor-induced increases in circulating proinflammatory cytokines (Bailey et al. 2011). Furthermore, *coprococcus* spp. was overrepresented in infants living with pets and was thought of as a potential bacterium supporting the hygiene hypothesis of preventing allergic diseases (Azad et al. 2013). Our results showed that *coprococcus* spp. significantly increased the via addition of zinc bacitracin in diets, suggested that zinc bacitracin can probably provide protection to intestinal tract. Considering those shifts in certain bacterial populations could be plausibly beneficial to healthy individuals, particularly if these bacterial populations are those affected by disease states (Ferrario et al. 2014). The above references suggest that the modulation of the *coprococcus* spp. by zinc bacitracin in the direction of potential protective microbiota can reduce the damage of stress and provide health protection on rex rabbits.

Antibiotic addition in the feed of animals can promote growth through the increased feed intake and weight gain (Cromwell 2002; Rettedal et al. 2009), which may be due to the microbiota alteration of the intestine (Rettedal et al. 2009). Furthermore, antibiotic improving animals health (e.g. prevent inflammation) through the modulated immune system is considered as a consequence of their impact on gut microbes (Willing et al. 2011; Ubeda and Pamer 2012; Brüssow 2015). Indeed, our study has the similar conclusions that antibiotics may promote the animal growth and the modulate inflammation.

In conclusion, our results demonstrated that chlortetracycline, colistin sulfate and zinc bacitracin supplementation have the tendency to improve the richness and diversity of rex rabbits caecum microorganism. Comparing three antibiotics, colistin sulfate and zinc bacitracin plays a better role than chlortetracycline in the promotion of animal growth and the prevention of disease. This information could guide the reasonable use and avoid the abuse of these three kinds of antibiotics of rex rabbits and serve for future studies on the design of antibiotics substitute or new nutritional strategies to promote rex rabbits growth and health. There lies a great controversy about the use of antibiotics in animal agriculture. Therefor, we need to pay more attention to the collateral effects of antibiotics. Moreover, it is necessary to further study the mechanism of antibiotics action on animals.

## Additional file

**Additional file 1.** Additional tables.

## Abbreviations

B: control
C: chlortetracycline
S: colistin sulfat
Z: zinc bacitracin
AGPs: antibiotics as growth promoters
OTUs: operational taxonomic units
PCoA: principal coordinate analysis
MRPP: multiresponse permutation procedure
GI: gastrointestinal

## References

[CR1] Abecia L, Fondevila M, Balcells J, Edwards JE, Newbold CJ, McEwan NR (2007). Effect of antibiotics on the bacterial population of the rabbit caecum. FEMS Microbiol Lett.

[CR2] Abella SR, Shelburne VB, Macdonald NW (2003). Multifactor classification of forest landscape ecosystems of Jocassee Gorges, southern Appalachian Mountains, South Carolina. Can J For Res.

[CR3] Andoh A, Fujiyama Y, Hata K, Araki Y, Takaya H, Shimada M, Bamba T (1999). Counter-regulatory effect of sodium butyrate on tumour necrosis factoralpha (TNF-alpha)-induced complement C3 and factor B biosynthesis in human intestinal epithelial cells. Clin Exp Immunol.

[CR4] Azad MB, Konya T, Maughan H, Guttman DS, Field CJ, Sears MR, Becker AB, Scott JA, Kozyrskyj AL (2013). Infant gut microbiota and the hygiene hypothesis of allergic disease: impact of household pets and siblings on microbiota composition and diversity. Allergy Asthma Clin Immunol.

[CR5] Bailey MT, Dowd SE, Galley JD, Hufnagle AR, Allen RG, Lyte M (2011). Exposure to a social stressor alters the structure of the intestinal microbiota: implications for stressor-induced immunomodulation. Brain Behav Immun.

[CR6] Bäuerl C, Collado MC, Zúñiga M, Blas E, Pérez MG (2014). Changes in cecal microbiota and mucosal gene expression revealed new aspects of epizootic rabbit enteropathy. Plos ONE.

[CR7] Brüssow H (2015). Growth promotion and gut microbiota: insights from antibiotic use. Environ Microbiol.

[CR8] Cani PD, Bibiloni R, Knauf C, Waget A, Neyrinck AM, Delzenne NM, Burcelin R (2008). Changes in gut microbiota control metabolic endotoxemia-induced inflammation in high-fat diet-induced obesity and diabetes in mice. Diabetes.

[CR9] Caporaso JG, Kuczynski J, Stombaugh J, Bittinger K, Bushman FD, Ek Costello, Fierer N, Peña AG, Goodrich JK, Gordon JI, Huttley GA, Kelley ST, Knights D, Koenig JE, Ley RE, Lozupone CA, McDonald D, Muegge BD, Pirrung M, Reeder J, Sevinsky JR, Turnbaugh PJ, Walters WA, Widmann J, Yatsunenko T, Zaneveld J, Knight R (2010). QIIME allows analysis of high-throughput community sequencing data. Nat Methods.

[CR10] Caporaso JG, Lauber CL, Walters WA, Berg-Lyons D, Huntley J, Fierer N, Owens SM, Betley J, Fraser L, Bauer M, Gormley N, Gilbert JA, Smith G, Knight R (2012). Ultra-high-throughput microbial community analysis on the Illumina HiSeq and MiSeq platforms. ISME J.

[CR11] Cho I, Yamanishi S, Cox L, Methé BA, Zavadil J, Li K, Gao Z, Mahana D, Raju K, Teitler I, Li H, Alekseyenko AV, Blaser MJ (2012). Antibiotics in early life alter the murine colonic microbiome and adiposity. Nature.

[CR12] Chung H, Pamp SJ, Hill JA, Surana NK, Edelman SM, Troy EB, Reading NC, Villablanca EJ, Wang S, Mora JR, Umesaki Y, Mathis D, Benoist C, Relman DA, Kasper DL (2012). Gut immune maturation depends on colonization with a host-specific microbiota. Cell.

[CR13] Cromwell CL (2002). Why and how antibiotics are used in swine production. Anim Biotechnol.

[CR14] Dao MC, Everard A, Aron-Wisnewsky J, Sokolovska N, Prifti E, Verger EO, Kayser BD, Levenez F, Chilloux J, Hoyles L, Dumas ME, Rizkalla SW, Doré J, Cani PD, Clément K, MICRO-Obes Consortium (2016). *Akkermansia muciniphila* and improved metabolic health during a dietary intervention in obesity: relationship with gut microbiome richness and ecology. Gut.

[CR15] Derrien M, Collado MC, Ben-Amor K, Salminen S, de Vos WM (2008). The mucin degrader *Akkermansia muciniphila* is an abundant resident of the human intestinal tract. Appl Environ Microbiol.

[CR16] Dibner JJ, Richards JD (2005). Antibiotic growth promoters in agriculture: history and mode of a ction. Poult Sci.

[CR17] Dubourg G, Lagier JC, Robert C, Armougom F, Hugon P, Metidji S, Dione N, Dangui NP, Pfleiderer A, Abrahao J, Musso D, Papazian L, Brouqui P, Bibi F, Yasir M, Vialettes B, Raoult D (2014). Culturomics and pyrosequencing evidence of the reduction in gut microbiota diversity in patients with broad-spectrum antibiotics. Int J Antimicrob Agents.

[CR18] Everard A, Belzer C, Geurts L, Ouwerkerk JP, Druart C, Bindels LB, Guiot Y, Derrien M, Muccioli GG, Delzenne NM, de Vos WM, Cani PD (2013). Cross-talk between *Akkermansia muciniphila* and intestinal epithelium controls diet-induced obesity. PNAS.

[CR19] Faith JJ, Guruge JL, Charbonneau M, Subramanian S, Seedorf H, Goodman AL, Clemente JC, Knight R, Heath AC, Leibel RL, Rosenbaum M, Gordon JI (2013). The long-term stability of the human gut microbiota. Science.

[CR20] Feighner SD, Dashkevicz MP (1987). Subtherapeutic levels of antibiotics in poultry feed and their effects on weight gain, feed efficiency, and bacterial cholyltaurine hydrolase activity. Appl Environ Microbiol.

[CR21] Ferrario C, Taverniti V, Milani C, Fiore W, Laureati M, Noni ID, Stuknyte M, Chouaia B, Riso P, Guglielmetti S (2014). Modulation of fecal clostridiales bacteria and butyrate by probiotic intervention with lactobacillus *paracasei* DG varies among healthy adults. J Nutr.

[CR22] Furet JP, Kong LC, Tap J, Poitou C, Basdevant A, Bouillot JL, Mariat D, Corthier G, Doré J, Henegar C, Rizkalla S, Clément K (2010). Differential adaptation of human gut microbiota to bariatric surgery-induced weight loss: links with metabolic and low-grade inflammation markers. Diabetes.

[CR23] Gu SH, Chen DD, Zhang JN, Lv XM, Wang K, Duan LP, Nie Y, Wu XL (2013). Bacterial community mapping of the mouse gastrointestinal tract. Plos ONE.

[CR24] Gulino LM, Ouwerkerk D, Kang AY, Maguire AJ, Kienzle M, Klieve AV (2013). Shedding light on the microbial community of the macropod foregut using 454-amplicon pyrosequencing. Plos ONE.

[CR25] Hippe B, Remely M, Bartosiewicz N, Riedel M, Nichterl C, Schatz L, Pummer S, Haslberger A (2014). Abundance and diversity of GI microbiota rather than IgG4 levels correlate with abdominal inconvenience and gut permeability in consumers claiming food intolerances. Endocr Metab Immune Disord Drug Targets.

[CR26] Injac R, Mlinaric A, Djorjevic-Milic V, Karljikovic- Rajic K, Strukelj B (2008). Optimal condition for determination of zinc bacitracin, polymyxin B, oxytetracycline and sulfacetamide in animal feed by micellar electrokinetic capillary chromatography. Food Addit Contam.

[CR27] Ismail NA, Ragab SH, Elbaky AA, Shoeib AR, Alhosary Y, Fekry D (2010). Frequency of Firmicutes and Bacteroidetes in gut microbiota in obese and normal weight Egyptian children and adults. Arch Med Sci.

[CR28] Jami E, White BA, Mizrahi I (2014). Potential role of the bovine rumen microbiome in modulating milk composition and feed efficiency. Plos ONE.

[CR29] Jones RL (2000). Clostridial enterocolitis. Vet Clin North Am Equine Pract.

[CR30] Jost T, Lacroix C, Braegger CP, Rochat F, Chassard C (2014). Vertical mother-neonate transfer of maternal gut bacteria via breastfeeding. Environ Microbiol.

[CR31] Kim BS, Jeon YS, Chun J (2013). Current status and future promise of the human microbiome. Pediatr Gastroenterol Hepatol Nutr.

[CR32] Lepage P, Leclerc MC, Joossens M, Mondot S, Blottière HM, Raes J, Ehrlich D, Doré J (2013). A metagenomic insight into our gut’s microbiome. Gut.

[CR33] Ley RE, Turnbaugh PJ, Klein S, Gordon JI (2006). Microbial ecology: human gut microbes associated with obesity. Nature.

[CR34] Liou AP, Paziuk M, Luevano JM, Machineni S, Turnbaugh PJ, Kaplan LM (2013). Conserved shifts in the gut microbiota due to gastric bypass reduce host weight and adiposity. Sci Transl Med.

[CR35] Louis P, Flint HJ (2007). Development of a semiquantitative degenerate real-time pcr-based assay for estimation of numbers of butyryl-coenzyme A (CoA) CoA transferase genes in complex bacterial samples. Appl Environ Microbiol.

[CR36] Lozupone C, Knight R (2005). UniFrac: a new phylogenetic method for comparing microbial communities. Appl Environ Microbiol.

[CR37] Mackie RI, Aminov RI, Hu W, Klieve AV, Ouwerkerk D, Sundset MA, Kamagata Y (2003). Ecology of uncultivated *Oscillospira* species in the rumen of cattle, sheep, and reindeer as assessed by microscopy and molecular approaches. Appl Environ Microbiol.

[CR38] Nam YD, Jung MJ, Roh SW, Ms Kim, Bae JW (2011). Comparative analysis of Korean human gut microbiota by barcoded pyrosequencing. Plos ONE.

[CR39] Neumann AP, Suen G (2015). Differences in major bacterial populations in the intestines of mature broilers after feeding virginiamycin or bacitracin methylene disalicylate. J Appl Microbiol.

[CR40] Niewold TA (2007). The nonantibiotic anti-inflammatory effect of antimicrobial growth promoters, the real mode of action? A hypothesis. Poult Sci.

[CR41] Panda S, Elkhader I, Casellas F, López VJ, García CM, Santiago A, Cuenca S, Guarner F, Manichanh C (2014). Short-term effect of antibiotics on human gut microbiota. Plos ONE.

[CR42] Pedersen R, Ingerslev HC, Sturek M, Alloosh M, Cirera S, Christoffersen BØ, Moesgaard SG, Larsen N, Boye M (2013). Characterisation of gut microbiota in Ossabaw and Göttingen minipigs as models of obesity and metabolic syndrome. Plos ONE.

[CR43] Peng ZR, Zeng D, Wang Q, Niu LL, Ni XQ, Zou FQ, Yang MY, Sun H, Zhou Y, Liu Q, Yin ZQ, Pan KC, Jing B (2016). Decreased microbial diversity and *Lactobacillus* group in the intestine of geriatric giant pandas (*Ailuropoda melanoleuca*). World J Microbiol Biotechnol.

[CR44] Place RF, Noonan EJ, Giardina C (2005). HDAC inhibition prevents NF-kappa B activation by suppressing proteasome activity: down-regulation of proteasome subunit expression stabilizes I kappa B alpha. Biochem Pharmacol.

[CR45] Poduval RD, Kamath RP, Corpuz M, Norkus EP, Pitchumoni CS (2000). Clostridium difficile and vancomycin-resistant enterococcus: the new nosocomial alliance. Am J Gastroenterol.

[CR46] Qin JJ, Li RQ, Raes J, Arumugam M, Burgdorf KS, Manichanh C, Nielsen T, Pons N, Levenez F, Yamada T, Mende DR, Li JH, Xu JM, Li SC, Li DF, Cao JJ, Wang B, Liang HQ, Zheng HS, Xie YL, Tap J, Lepage P, Bertalan M, Batto JM, Hansen T, Paslier DL, Linneberg A, Nielsen H, Pelletier E, Renault P, SicheritzPonten T, Turner K, Zhu HM, Yu C, Li ST, Jian M, Zhou Y, Li YR, Zhang XQ, Li SG, Qin N, Yang HM, Wang J, Brunak S, Doré J, Guarner F, Kristiansen K, Pedersen O, Parkhill J, Weissenbach J, Consortium M, Bork P, Ehrlich S, Wang J (2010). A human gut microbial gene catalog established by metagenomic sequencing. Nature.

[CR47] Rettedal E, Vilain S, Lindblom S, Lehnert K, Scofield C, George S, Clay S, Kaushik RS, Rosa AJ, Francis D, Brözel VS (2009). Alteration of the ileal microbiota of weanling piglets by the growth-promoting antibiotic chlortetracycline. Appl Environ Microbiol.

[CR48] Rodriguez AV, Nadra MCMD (1995). Effect of pH and hydrogen peroxide produced by *Lactobacillus hilgardii* on *Pediococcus pentosaceus* growth. FEMS Microbiol Lett.

[CR49] Shin NR, Whon TW, Bae JW (2015). Proteobacteria: microbial signature of dysbiosis in gut microbiota. Trends Biotechnol.

[CR50] Thompson JR, Marcelino LA, Polz MF (2002). Heteroduplexes in mixed-template amplifications: formation, consequence and elimination by ‘reconditioning PCR’. Nucleic Acids Res.

[CR51] Tims S, Derom C, Jonkers DM, Vlietinck R, Saris WH, Kleerebezem M, de Vos WM, Zoetendal EG (2013). Microbiota conservation and BMI signatures in adult monozygotic twins. ISME J.

[CR52] Torok VA, Allison GE, Percy NJ, Ophel-Keller K, Hughes RJ (2011). Influence of antimicrobial feed additives on broiler commensal posthatch gut microbiota development and performance. Appl Environ Microbiol.

[CR53] Ubeda C, Pamer EG (2012). Antibiotics, microbiota, and immune defense. Trends Immunol.

[CR54] Velkov T, Thompson PE, Nation RL, Li J (2010). Structure—activity relationships of polymyxin antibiotics. J Med Chem.

[CR55] Wang Q, Garrity GM, Tiedje JM, Cole JR (2007). Naїve bayesian classifier for rapid assignment of rRNA sequences into the new bacterial taxonomy. Appl Environ Microbiol.

[CR56] Willing BP, Russell SL, Finlay BB (2011). Shifting the balance: antibiotic effects on host-microbiota mutualism. Nat Rev Microbiol.

[CR57] Zeng B, Han SS, Wang P, Wen B, Jian WS, Guo W, Yu ZJ, Du D, Fu XC, Kong FL, Yang MY, Si XH, Zhao JC, Li Y (2015). The bacterial communities associated with fecal types and body weight of rex rabbits. Sci Rep.

[CR58] Zhang C, Li S, Yang L, Huang P, Li W, Wang S, Zhao G, Zhang M, Pang X, Yan Z, Liu Y, Zhao L (2013). Structural modulation of gut microbiota in life-long calorie-restricted mice. Nat Commun.

[CR59] Zhu Y, Wang C, Li F (2015). Impact of dietary fiber/starch ratio in shaping caecal microbiota in rabbits. Can J Microbiol.

