# Supplementary material for: Illumina Miseq platform analysis caecum bacterial communities of rex rabbits fed with different antibiotics
Source: AMB Express. 2016 Oct 21;6:100. doi: 10.1186/s13568-016-0273-1 (PMC5074941; doi:10.1186/s13568-016-0273-1)
Supplement: Supplementary file 1 — Additional file 1. Additional tables. [file 13568_2016_273_MOESM1_ESM.docx]

Table S1 OTUs table summary

| Sample name | Sequence counts | Average reads |
| --- | --- | --- |
| B1 | 43,598 | 253 |
| B2 | 9,043 | 253 |
| B3 | 39,293 | 253 |
| B4 | 39,057 | 253 |
| B5 | 44,869 | 253 |
| C1 | 11,080 | 253 |
| C2 | 18,391 | 252 |
| C3 | 55,909 | 253 |
| C4 | 43,074 | 253 |
| C5 | 45,661 | 253 |
| S1 | 42，650 | 253 |
| S2 | 25,665 | 253 |
| S3 | 42,631 | 253 |
| S4 | 177,285 | 253 |
| S5 | 20,654 | 253 |
| Z1 | 27,580 | 253 |
| Z2 | 200,735 | 253 |
| Z3 | 29,621 | 253 |
| Z4 | 23,574 | 253 |
| Z5 | 21,886 | 253 |

In the table B, C, S, Z represent of control, chlortetracycline, colistin sulfate and zinc bacitracin, respectively.

Table S2 Phylum level were identified microflora

| Microbial name | Control | Chlortetracycline | Colistin sulfate | Zinc bacitracin |
| --- | --- | --- | --- | --- |
| Firmicutes | 0.643055006 | 0.582139559 | 0.66051972 | 0.696055929 |
| Bacteroidetes | 0.204379369 | 0.246326342 | 0.174699908 | 0.151985226 |
| Verrucomicrobia | 0.04659016 | 0.024825221 | 0.029969661 | 0.022661918 |
| Proteobacteria | 0.032027437 | 0.036749769 | 0.033926923 | 0.020788814 |
| Tenericutes | 0.032317636 | 0.034771138 | 0.034111595 | 0.036802533 |
| Crenarchaeota | 0 | 0.000026382 | 0.008969793 | 0.0130062 |
| Acidobacteria | 0 | 0.006410764 | 0.005830365 | 0.0033241 |
| Actinobacteria | 0.001582905 | 0.007835378 | 0.00105527 | 0.001846722 |
| Euryarchaeota | 0.003561535 | 0.001108033 | 0.000870598 | 0.000105527 |
| Chloroflexi | 0 | 0.00242712 | 0.000817834 | 0.000263817 |
| TM7 | 0.002743701 | 0.004801477 | 0.001688432 | 0.007334125 |
| Gemmatimonadetes | 0 | 0.00226883 | 0.00015829 | 0.000105527 |
| Cyanobacteria | 0.002453502 | 0.003640681 | 0.00393088 | 0.002400739 |
| Planctomycetes | 0 | 0.000184672 | 0.00211054 | 0.00105527 |
| Nitrospirae | 0 | 0.001582905 | 0.000316581 | 0.000184672 |
| Synergistetes | 0.000844216 | 0.000211054 | 0 | 0.000052763 |
| WS3 | 0 | 0.000474871 | 0.000079145 | 0.000052763 |
| NC10 | 0 | 0.000369344 | 0 | 0 |
| Chlorobi | 0 | 0.000290199 | 0.000026382 | 0 |
| AD3 | 0 | 0.000184672 | 0 | 0 |
| OP8 | 0 | 0.000079145 | 0 | 0 |
| SBR1093 | 0 | 0.000052763 | 0.000052763 | 0 |
| Armatimonadetes | 0 | 0.000052763 | 0.000026382 | 0 |
| NKB19 | 0 | 0 | 0 | 0.000026382 |
| WPS-2 | 0 | 0.000026382 | 0 | 0 |
| BRC1 | 0 | 0 | 0 | 0.000026381 |
| Others | 0.030444532 | 0.043160533 | 0.040838939 | 0.041920591 |
